# Privacy, Security, and Legal Issues in the Health Cloud: Structured Review for Taxonomy Development

**DOI:** 10.2196/38372

**Published:** 2024-02-12

**Authors:** Zahra Zandesh

**Affiliations:** 1 Information Technology and Statistics Department Tehran University of Medical Sciences Tehran Iran

**Keywords:** taxonomy, privacy, security, legal, cloud computing

## Abstract

**Background:**

Privacy in our digital world is a very complicated topic, especially when meeting cloud computing technological achievements with its multidimensional context. Here, privacy is an extended concept that is sometimes referred to as legal, philosophical, or even technical. Consequently, there is a need to harmonize it with other aspects in health care in order to provide a new ecosystem. This new ecosystem can lead to a paradigm shift involving the reconstruction and redesign of some of the most important and essential requirements like privacy concepts, legal issues, and security services. Cloud computing in the health domain has markedly contributed to other technologies, such as mobile health, health Internet of Things, and wireless body area networks, with their increasing numbers of embedded applications. Other dependent applications, which are usually used in health businesses like social networks, or some newly introduced applications have issues regarding privacy transparency boundaries and privacy-preserving principles, which have made policy making difficult in the field.

**Objective:**

One way to overcome this challenge is to develop a taxonomy to identify all relevant factors. A taxonomy serves to bring conceptual clarity to the set of alternatives in in-person health care delivery. This study aimed to construct a comprehensive taxonomy for privacy in the health cloud, which also provides a prospective landscape for privacy in related technologies.

**Methods:**

A search was performed for relevant published English papers in databases, including Web of Science, IEEE Digital Library, Google Scholar, Scopus, and PubMed. A total of 2042 papers were related to the health cloud privacy concept according to predefined keywords and search strings. Taxonomy designing was performed using the deductive methodology.

**Results:**

This taxonomy has 3 layers. The first layer has 4 main dimensions, including cloud, data, device, and legal. The second layer has 15 components, and the final layer has related subcategories (n=57). This taxonomy covers some related concepts, such as privacy, security, confidentiality, and legal issues, which are categorized here and defined by their expansion and distinctive boundaries. The main merits of this taxonomy are its ability to clarify privacy terms for different scenarios and signalize the privacy multidisciplinary objectification in eHealth.

**Conclusions:**

This taxonomy can cover health industry requirements with its specifications like health data and scenarios, which are considered as the most complicated among businesses and industries. Therefore, the use of this taxonomy could be generalized and customized to other domains and businesses that have less complications. Moreover, this taxonomy has different stockholders, including people, organizations, and systems. If the antecedent effort in the taxonomy is proven, subject matter experts could enhance the extent of privacy in the health cloud by verifying, evaluating, and revising this taxonomy.

## Introduction

### Background

Cloud computing is among the hottest core technical topics in the digital world. It has broad-ranging effects across IT, business, software engineering, and data storage. One of the main effects is an increase in capability. According to the National Institute of Standards and Technology (NIST) definition, “cloud computing is a model for enabling convenient, resource pooling, ubiquitous, on-demand access which can be easily delivered with different types of service provider interactions” [1,2]. Cloud technology can meet the requirements of the health care industry. It has some benefits like helping health organizations to reduce their costs by replacing and migrating all IT infrastructure, platforms, and software to the cloud, and providing integrated services across multiple organizations with delivery of better access to IT knowledge, resources, and services in a more technical and economical way.

The cloud in the health care context can increase medical record accessibility and make medical history available for individuals. Moreover, it can enhance cooperation among various stakeholders in the health industry through the integration of electronic medical information from dispersed locations and can reduce medical error complications to achieve patients’ lifesaving goals [3-9]. A health record includes a chronological account of an individual’s tests and treatments, and is a critical part of any health care lawsuit about health care procedures [10-14]. These documents can play an important role in guarding individuals based on medical ethics concerns, patients’ rights, and the bill of rights in each country [15-18]. Therefore, acceptance of any kind of computing technology with the combination of medical informatic applications can change the boundaries of health care organizations [1].

Despite all these benefits, the sharing and storing of sensitive electronic health data and personal health information through the cloud raise various privacy and security concerns [2,3]. An important concern is the probable release of health information to third parties who are not authorized to access the information. The distributed architecture of the cloud causes many difficulties like service accessibility, data reliability, data management, scalability, interoperability, privacy, security, data ownership, regulation and standards, organizational change, business process reengineering, etc [3-8].

The tradeoffs between the pros and cons of this technology depend on the approaches that governments introduce to address the privacy, security, and legal challenges in such a complicated domain like health care.

The challenges are magnified several times when there are no definite implications for some essential and technical concepts. For example, privacy in the digital world is a term with different meanings, which can clearly include a wide range of concepts and can completely differ from its traditional comprehension [3]. Moreover, some interpreters have explained this word as “vague and evanescent” [4]. Therefore, a lack of transparency in the privacy concept has made policy making difficult [19-21].

In these occasions, judges and legislators cannot obviously speak about privacy harms, especially at intersections with other fields like free speech, effective consumer transactions, and security, which are quite controversial. It is completely understandable that privacy and the related implications are complex and multidimensional, and are thus considered legal, philosophical, or even technical. 

Furthermore, the involute definitions of privacy and cloud technological risks have stopped governments from adopting cloud technology in the health industry, and if cloud technologies are introduced in the health industry, issues like security, privacy, and legal obstacles play preventive roles. In other words, using cloud capabilities in the health industry without proper setups can lead to disastrous outcomes, such as blackmail and threats. As the relationship between the growth of eHealth and privacy value is quite obvious, it is necessary to create a balance between the pros and cons of these technologies in this new era. Health care stakeholders in different countries have taken many efforts to identify political and legal challenges in this domain and have developed appropriate supplements and technological infrastructure for the health cloud [22-24]. Moreover, the obstacles have led them to revise and redesign required concepts to make them compatible for the new paradigm [13,14,16,17].

A review of previous taxonomies appears necessary to obtain a better overall view. The most popular and famous taxonomies in this domain were analyzed by their features and attributes. The goals, use, and dimensions of each taxonomy in the privacy era are presented in Table 1.

Almost all reports in Table 1 declared that privacy is a multilateral concept that needs analysis from different sides. In addition, the reports indicated that the data value has grown incredibly, which could be the most valuable asset for organizations and individuals, but privacy-preserving concerns were illustrated as nonignorable challenges. Some reports only dealt with security services and presented those as privacy matters, while others only paid attention to legal issues or data features. Obviously, most of them were not specifically designed based on cloud technology features or health care scenarios.

**Table 1:** Previous taxonomies.

Reference	Title	Goals	Dimensions
Barker et al [25]	Data privacy taxonomy	This taxonomy was designed for privacy features and had 4 dimensions, each of which had their own subcategories and demonstrated their relationships in data repositories, such as database management systems, which are used for data mining.	Purpose, Visibility, Granularity, and Retention
Antón et al [26]	Taxonomy of privacy requirements for web sites	The authors analyzed websites to design an internet privacy policy taxonomy for goal mining and extraction of prerequirement goals from postrequirement text artifacts. The goals of privacy in this work are classified as privacy protection and privacy vulnerabilities.	Privacy protection goals: Notice and awareness, Choice and consent, Access and participation, Integrity and security, and Enforcement and redress; Privacy vulnerabilities: Monitoring, Aggregation, Storage, and Transfer of information phases
Asaddok et al [27]	Usability, security, and privacy taxonomy for mobile health applications	This taxonomy provided a model for mobile health applications, which were identified based on a study on products on the market. It had 3 dimensions, and each of them had their own subcategories (overall 10).	Usability, Security, and Privacy
Heurix et al [28]	Taxonomy for privacy-enhancing technologies	This taxonomy was designed to provide a classification method owing to the various features of privacy-enhancing technologies. The purpose was to cover various techniques, such as anonymization or encryption, with different application scenarios. Each of its dimensions had its own subsets.	Scenario, Aspect, Aim, Foundation, Data, Trusted third party, and Reversibility
Kotz [29]	Threat taxonomy for mobile health privacy	This work presented a taxonomy for mobile health privacy and emphasized mobility and networking with many risks. There was a focus on the effects that threats could have, and threats were organized based on their type.	Misuse of patient identities, Unauthorized access or modification of PHI^a^, and Disclosure of PHI
Skinner et al [30]	Information privacy taxonomy for collaborative environments	This taxonomy had 3 dimensions, and each dimension was interrelated and had different influences over information privacy. These dimensions translated into 3 corresponding views of information privacy within a collaborative environment, like computation view, content view, and structural view.	Time, Matter, and Space
Stein [31]	Taxonomy of privacy	This work organized all kinds of harms and is one of the most well-known taxonomies in the field. Four different types of harmful activities covered by privacy were identified. Each activity type had its subactivities (n=16).	Information collection, Information processing, Information dissemination, and Invasion
Vatsalan et al [32]	Taxonomy of privacy-preserving record linkage techniques	Privacy-Preserving Record Linkage taxonomy is another study that provides an overview of techniques that allow linking of databases among organizations. These techniques provide privacy preservation at the same time.	Privacy aspects, Linkage techniques, Theoretical analysis, Evaluation, and Practical aspects
Zandesh et al [3]	Legal framework for a health cloud	This work was a systematic review that introduced a legal framework for the health cloud with 5 main pillars and 17 subcomponents, and defined the role of legal aspects in the reliability of eHealth.	Compliance, Data protection, Identity credential access management, Ownership, and Quality of service
Olla et al [33]	Mobile health taxonomy	This taxonomy had 8 categories under 3 main pillars owing to the application’s intended purpose.	Medical use cases, Technical modalities, and Consideration
Association for Computing Machinery [34]	Computing classification system from the ACM^b^	This taxonomy was developed to organize papers received in the ACM Digital Library or events hosted by the ACM.	Cryptography, Formal methods and theory of security, Security services, Intrusion/anomaly detection and malware mitigation, Security in hardware, System security, Network security, Database and storage security, Human and societal aspects of security and privacy, and Software and application security
Computer Security Division/NIST^c^ [35]	Computer security resource center classification from the NIST	This classification was a significant reference for cybersecurity considerations that provided a comprehensive model for cybersecurity knowledge.	Security and privacy-specific research domains, Technologies, Applications, Laws and regulations, Types of activities, and Business sectors
IEEE^d^ [36]	IEEE taxonomy	IEEE developed a taxonomy to organize papers received in IEEE Xplore Digital Library or events hosted by IEEE.	Access control, Computer security, Cryptography, Data security, Information security, and Terrorism
ETSI^e^ [37]	ETSI	This institute organized a technical committee to improve the level of privacy and security for European organizations and citizens in Europe and across the world by standard development. In general, ETSI provided an overview of the global cyber security ecosystem.	Cybersecurity, Securing technologies and systems, and Security tools and techniques
IFIP^f^ [38,39]	Technical committees of the IFIP	This independent organization covered working groups or committees on information processing. Among the committees, one of its technical committees has worked on security and privacy protection in information processing systems. The product of this committee provided the most extensive collection of concepts and topics. However, generally, this report could not be considered as a taxonomy.	Information security management, General system security, Data and application security and privacy, Network and distributed system security, IT assurance and audit, Identity management, IT misuse and the law, Information security education, Digital forensics, Critical infrastructure protection, Trust management, Human aspects of information security and assurance, Information system security research, and Secure engineering
Federal Office for Information Security [40]	IT baseline protection methodology from the German Federal Office	This methodology has developed a catalog to support information security and the development of cybersecurity evaluation in organizations.	General aspects, Infrastructure, IT systems, Networks, and IT applications
Nai Fovino et al [41]	Taxonomy of the Joint Research Center from the European Commission’s science and knowledge service	The main goal of this taxonomy was aligning cybersecurity terminologies, definitions, and domains to facilitate EU cybersecurity competency categorization. It included 3 completely intertwined dimensions to provide evidence-based scientific support to the European policy-making process.	Cybersecurity domains, Sectors, and Applications and technologies

^a^PHI: personal health information.

^b^ACM: Association for Computing Machinery.

^c^NIST: National Institute of Standards and Technology.

^d^IEEE: Institute of Electrical and Electronics Engineers.

^e^ETSI: European Telecommunications Standards Institute.

^f^IFIP: International Federation for Information Processing.

### Problem Statement

Despite all previous studies, it appears that more efforts are needed to redefine the privacy concept in the health domain, especially in the cloud context. The nomenclature and classification confusion in privacy terminology prevent businesses from finding a comprehensive solution for the domain requirements [22-24]. It is worthwhile to note that taxonomy use is an effective approach. Regarding the research question, our attempts focus on reaching a comprehensive concept about privacy.

The main challenges are related to what we already know and what we need to know. Therefore, a clear and precise taxonomy would be helpful to identify the specifications of privacy in a dynamic environment and would help in conducting future research projects for evaluating its impacts. A taxonomy was developed in this study, and the study contributions are presented below.

### Study Contributions and Objectives

This study has several implications. It redefines privacy with regard to the health cloud and focuses on identifying the main approaches to deal with the contributed factors and dimensions that rely on taxonomy designing.

This taxonomy clarifies the privacy concept in eHealth, which is a multidisciplinary context, and tries to eliminate the ambiguity of this subject in cloud environments with regard to the different requirements in health care scenarios and situations.

The proposed taxonomy provides a true and complete perspective regarding the intervention, management, and handling of other variables, as well as the itemization of the expected outcomes and the determination of how best to assess them, thus clarifying the units of analysis in health cloud privacy research.

The findings of this study regarding the privacy taxonomy led to the distinction and clarification of the overlapping and vague structure of related concepts, and privacy was defined by identifying the discrete sets of variables representing specific privacy configurations and definitive boundaries for “security,” “privacy,” and “legal” terms, which are crucial for future research, policy making, and the actual management of privacy. This capability of the taxonomy was considered as the main outcome or contribution of this study, and it conceptually provides quite clear boundaries of these terms in the digital health world.

The proposed taxonomy has 3 layers, of which the first layer has 4 main dimensions, including cloud, data, device, and legal, and the second layer has 15 components, with each of them having subcomponents (n=57). This taxonomy has some advantages like presenting the hierarchical root of concepts and the inherited features of taxonomies. The specific implementation was performed by selecting published English papers related to the concept of health cloud privacy from several databases and relying on predefined keywords and search strings, followed by a classification design through a qualitative content analysis approach.

Hence, this taxonomy could cover health industry requirements with its specifications like health data and scenarios, which are considered to be the most complicated among businesses and industries. Therefore, this taxonomy could be generalized to other domains and businesses with less complications.

Previous taxonomies in the privacy domain have also been covered in this article, and the designing steps of the new taxonomy are presented in the Methods section.

## Methods

### Methodology Analysis

One of the main concerns in various disciplines is how to group disciplines based on taxonomies. Such a classification has given taxonomies a pivotal role for researchers and practitioners in investigations and businesses as it has enabled them to comprehend and analyze complex domains [42,43].

Covering both descriptive knowledge and prescriptive knowledge, design science also consists of taxonomies as a type of conceptual knowledge in its epistemology. The research goal at the conceptual level is essentialist: concepts and conceptual frameworks at this level aim at identifying essences in the research territory and their relationships [44].

The term taxonomy is different from other similar words. Compared with classification, in some literature, it refers to groupings that are derived based on empirical studies with involvement of cluster analysis and statistical techniques. This definition is also referred to as numerical taxonomy [45].

Taxonomy is also considered as a classification scheme [46], and it is possible to use the terms of classification scheme, taxonomy, and typology as substitutes of each other. A previous report mentioned 3 approach categories for taxonomy: inductive, deductive, and intuitive [43].

With respect to the inductive approach, empirical cases are taken into account. In the following step, they are analyzed so as to realize dimensions and characteristics in the taxonomy. In this type of analysis, a variety of statistical techniques, such as cluster analysis, or other less rigorous techniques are employed [47].

In the deductive approach, the taxonomy involves theory or conceptualization rather than empirical cases. The method uses a logical process that results from a sound conceptual or theoretical foundation in order to clarify dimensions and characteristics in the taxonomy. It is considered to be similar to the cladistics approach in biology [47]. The method may involve an analysis of empirical cases so that evaluation or even modification of the taxonomy can be performed.

The intuitive approach is considered in the case of necessity. The objects are categorized based on what a researcher comprehends. In this approach, the taxonomy is offered on the basis of the perceptions of a researcher. This technique is not explicitly used [47].

Our proposed privacy taxonomy is derived by the deductive approach. Thematic analysis, which is often called qualitative content analysis, is considered as the methodology for the implementation of the deductive approach and as one of the most favorable methodologies in taxonomy creation [19]. Content analysis, as a research method, is a systematic and objective means of describing and quantifying phenomena. It is also known as a method for analyzing documents. This research method is used for making replicable and valid inferences from the data to their context, with the purpose of providing knowledge, new insights, a representation of facts, and a practical guide to action. In most cases, those concepts or categories are applied to construct models, conceptual systems, conceptual maps, or categories [20].

This type of taxonomy development needs a complete literature review like a systematic or structured review because a systematic review relies on the following: definite time, definite inclusion criteria, definite information sources, and structured study selection according to predefined PRISMA (Preferred Reporting Items for Systematic Reviews and Meta-Analyses) guidelines. This method has been described in the Taxonomy Development Characteristics subsection.

### Ethics Approval

This study did not include human participants or animals, and thus, ethics approval was not required.

### Eligibility Criteria

Published English papers (inclusion criterion 1) related to privacy aspects in the health cloud (inclusion criterion 2) were used to create a privacy taxonomy for the health cloud.

### Information Sources

Designated databases, including Web of Science, IEEE Digital Library, Scopus, Google Scholar, and PubMed, were searched from April to June 2020 to identify relevant articles.

### Study Selection

Study selection involved the following 5 different phases:

1. Health and computer science databases were chosen to cover all related publications. This step was applied to papers after 2010.
2. “Health cloud,” “privacy,” “medical ethics,” “data management,” “compliance management,” and “medical devices” were the keywords considered with divergent MeSH (Medical Subject Headings) terms.
3. Different search strategies on keywords were adopted for each electronic database to obtain more relevant papers.
4. The identified papers were screened based on the eligibility criteria using their titles, abstracts, and keywords.
5. Papers not eliminated in the previous phase were read completely.

### Taxonomy Development Characteristics

The new taxonomy was developed on the basis of the deductive approach in 6 phases. The initial phase involved reading data intensively and assessing the papers. The second phase involved configuring the main dimensions to align with the research goals. This phase analyzed the results through Excel files. The third phase included data coding in main classes where the results were categorized. In the fourth phase, the main classes were structured and then arranged into components and subcomponents in an inductive manner, and subcomponents were designated to components. In the fifth phase, the results were categorically analyzed and then presented. The final phase involved reporting and documentation.

A total of 2042 papers were identified, of which 585 were discarded because of repetition in different databases (first layer of filtering according to inclusion criterion 1). The remaining 1457 papers were analyzed on the basis of their titles, abstracts, and keywords. Ultimately, the outcome was divided into 3 categories (second layer of filtering according to inclusion criterion 2).

**Figure 1:**
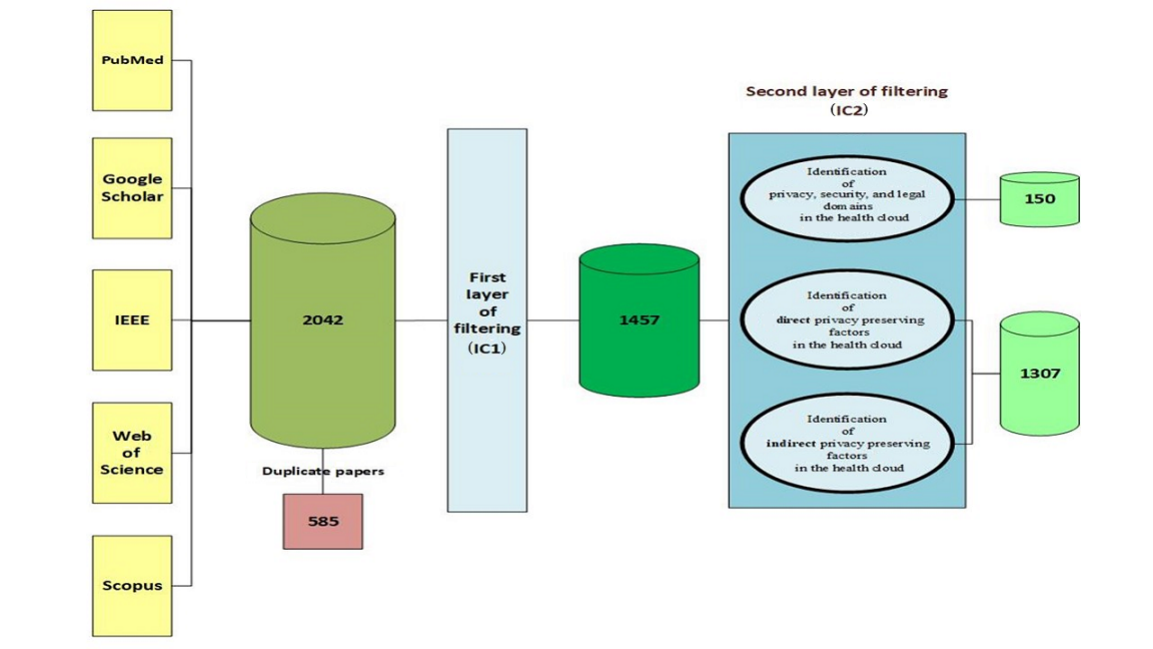
PRISMA (Preferred Reporting Items for Systematic Reviews and Meta-Analyses) flow diagram. IC: inclusion criterion.

In the second layer of filtering, initially, 150 papers were chosen according to the privacy, security, and legal domains in the health cloud, which were related to the first category of this work (Figure 1). By reading the full texts in this category, it can be judged that different headlines like compliance management, data management, data governance, information security services, medical ethics, patients’ rights, privacy issues, and technology considerations play important roles in privacy management discipline and influence privacy preservation in the health cloud environment. The identified domains provided a new map and road for the construction of the taxonomy of privacy. These domains led to the identification of probable dimensions, components, and subcategories in related contexts.

Subsequently, with the above-mentioned domains and according to the second layer of filtering (inclusion criterion 2), the rest of this work was conducted, which helped to group the 1307 remaining papers. The full texts of the papers were analyzed according to their details. The findings of the analysis phases showed that many related factors can influence privacy-preserving topics in the health cloud. Consequently, the identified factors were coded and grouped into direct and indirect groups for taxonomy creation, and they formed the second and third categories of the PRISMA guidelines. These factors influence privacy preservation in the health cloud. The findings of study selection are shown in a PRISMA flow diagram (Figure 1).

In this study, according to a previous report [43], attempts were made to cover all qualitative attributes, such as conciseness, robustness, comprehensiveness, extendibility, and explanatory ability. The aim was to develop a taxonomy based on a set of dimensions, with each including characteristics describing the objects comprehensively in a specific domain of interest.

Table 2 presents the 6 phases involving the formation and adoption of our taxonomy. The subsequent sections present a detailed introduction with respect to each dimension’s components and subcomponents. The privacy taxonomy can be provided in several different approaches, and hierarchical taxonomy is the most notable method. 

**Table 2:** Taxonomy development phases.

Phase	Thematic analysis method/qualitative content analysis method	Adoption in our work
1	Reading data intensively and assessing papers	A total of 1457 papers were identified from among 2042 papers. The papers were analyzed on the basis of their titles, abstracts, and keywords, and their security, privacy, and legal features were chosen.
2	Configuring the main dimensions to correspond to the goals of this paper	The full texts of selected results in the previous phase were analyzed and processed by their details in an Excel spreadsheet. The outcome was divided into 3 categories: The first category involves the identification of privacy, security, and legal domains in the health cloud, and 150 related papers were identified. The second and third categories involve the identification of direct and indirect factors that impact privacy preservation in the health cloud. A total of 1307 remaining papers were examined by their contents.
3	Data coding in main classes	The most frequent and important features were categorized into 76 analytical categories.
4	Structuring the main classes and configuring components and subcomponents inductively on the material, and assigning subcomponents to components	The analytical categories were then synthesized into the taxonomy. The taxonomy requires a multidimensional and hierarchical structure, and each tier in the hierarchy inherits all attributes of the tier immediately above it. The highest level in the hierarchy has the greatest generality and vice versa. The subcomponents may be used to improve the domain concept under consideration and the relationships between the nodes and leaves in the hierarchy. Iterative processes can lead to taxonomy constructors. The privacy taxonomy provides a heuristic representation of hierarchies with 4 dimensions of privacy and branches in each dimension. This model allows for more specification of independent variables in the model development and with regard to the research objectives.
5	Performing category-based analyses and presenting the results	The taxonomy has 3 layers, of which the first layer has 4 main dimensions, including cloud, data, device, and legal. The second layer has 15 components, and each of them has subcomponents (n=57). This well-organized taxonomy has some advantages like presenting the hierarchical root of concepts and the inherited features of taxonomies.
6	Reporting and documentation	Finally, the taxonomy was derived and proposed from the abstraction of each of the dimensions.

## Results

After analyzing the identified papers and considering taxonomy development, with respect to studies related to the first category of the method in the digital world, it was found that only documented rules and regulations did not comply with the privacy, security, and legal requirements in the health cloud. To be more precise, compliance alone cannot consider and resolve all the privacy, security, and legal requirements in such a dynamic environment like the cloud, and as mentioned before, some other headings like compliance management, data management, data governance, information security services, medical ethics, patients’ rights, privacy issues, and technology considerations play important roles. To cover all these domains and overcome previous deficiencies, a taxonomy of privacy, security, and legal issues in the health cloud was designed.

**Figure 2:**
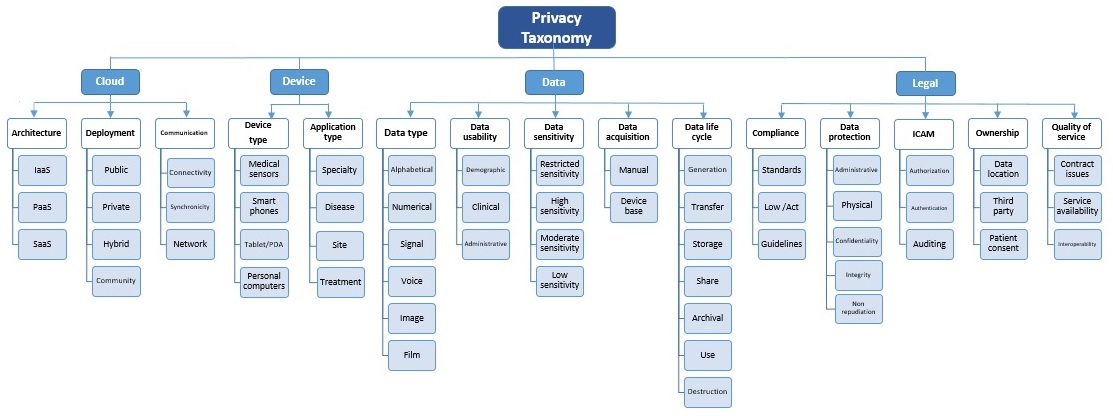
Proposed taxonomy of privacy in the health cloud. IaaS: infrastructure as a service; ICAM: identity credential access management; PaaS: platform as a service; PDA: personal digital assistant; SaaS: software as a service.

As illustrated in Figure 2, this taxonomy has 3 layers. Different features in this context were initially grouped into 4 dimensions, namely the cloud specification, legal aspect, data specification, and device specification in the context of privacy. This classification provided the first or most comprehensive level of generality in the taxonomy of privacy. Other factor identification was related to the next level of taxonomy, and the second and third levels of taxonomy creation and identification led to the introduction of direct and indirect factors for privacy preservation. Then, the basic building blocks or dimensions, components, and subcategories were realized with a qualitative content analysis. The second layer identified 15 components, with each of them having subcomponents (n=57). This model allows for more specification of independent variables in model development and with regard to research objectives.

**Figure 3:**
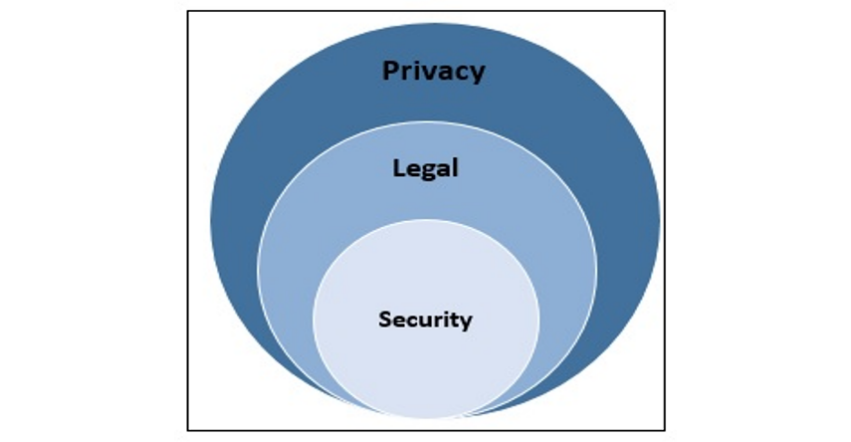
Definitive boundaries between “security,” “legal,” and “privacy” in digital health.

The findings of this paper helped to process and define privacy by identifying a composite set of variables that represent to the extent possible the true nature of interventions and by incorporating the major dimensions of privacy and their constituent parts. Moreover, the findings led to the creation of a new conceptual diagram, which has been presented in Figure 3. The main outcomes or results of this taxonomy appear in this figure, which provides a definite boundary for each of the ambiguous terms like privacy, legal, and security. This figure displays conceptual coverage and overlapping boundaries of these terms in the digital health world, which are crucial for future research, policy making, and the actual management of privacy.

According to the proposed taxonomy, each circle has its subdomains. In the Discussion section, each dimension’s components and subcomponents are introduced in detail.

## Discussion

### Principal Findings

The details of each dimension’s components and subcomponents (Figure 2) are provided. The main characteristics included in the taxonomy are described and discussed to answer the research question, and an attempt was made to focus on reaching a comprehensive concept regarding privacy.

The question is as follows: Which dimensions and factors affect privacy taxonomy and should be considered in current health cloud projects or systems for privacy preservation?

As mentioned in the Results section, to provide a clear and precise taxonomy according to the method steps, selected papers were studied and analyzed deeply, which led to 4 new dimensions, namely cloud, legal, data, and device. All these dimensions were related to privacy specifications.

In the below sections, each dimension of the proposed taxonomy, and its components and subcomponents are described extensively to provide better understanding for audiences.

### Implications

#### Dimension 1: Cloud

The first dimension of this taxonomy is the cloud, which incorporates all aspects of cloud computing technology. It is an evolving paradigm that is useful in the health care context and has an indirect impact on privacy. The cloud dimension has 3 main characteristics, each of which has its specialty: architecture, deployment, and communication. According to the NIST definition, the cloud can be defined based on its characteristics as follows: an architecture or service model, which is defined based on its limited taxonomy, and it can also be defined based on its deployment model with service delivery or business operation, which can affect its features [48]. It is worthwhile to mention that each state of these components will affect the privacy of information in the cloud, which cannot be ignored.

**Figure 4:**
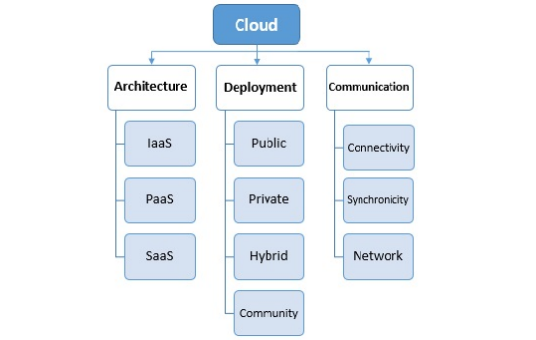
Components of the cloud dimension. IaaS: infrastructure as a service; PaaS: platform as a service; SaaS: software as a service.

Furthermore, several methods of communication can be defined between the cloud providers and the cloud customers in the cloud. Each of them contains characteristics having an indirect effect on privacy. These aspects are grouped into 3 parts in Figure 4, with each containing subcomponents.

##### Architecture or Service Model

There are several service models defined for the cloud, and their subcomponents constitute the first component of the cloud dimension [48-51].

Software as a service (SaaS) enables the client to receive services from applications where providers use cloud services to provide the services. It is important to note that the client cannot manage and control the cloud infrastructure, including networks, servers, operating systems, and storage, or even individual application capabilities.

Platform as a service (PaaS) enables the client to provide services on the cloud through consumer-created or acquired applications created using some programming languages, libraries, services, and tools in the cloud. The difference is that the client no longer manages and controls the cloud infrastructure, including networks, servers, operating systems, and storage, or even individual application capabilities. Hence, the client only controls the executed application and the configuration settings for the application-hosting environment.

Infrastructure as a service (IaaS) enables the client to provide processing, storage, network, and other fundamental computing resources, where the client can deploy and run arbitrary software including operating systems and applications. The client does not manage or control the underlying cloud infrastructure and has control over operating systems, storage, and deployed applications and possibly limited control of select networking components.

##### Deployment Model

The second subcategory of this component is the cloud deployment model [48,49].

Private cloud is used by a single organization that has different consumers and stakeholders. This infrastructure may be administered or handled by that organization, a third party, or their combination and may exist on or off the premises.

Community cloud is used by a specific community of consumers from organizations with shared concerns. This infrastructure may be administered or handled by one or more organizations in the community, a third party, or their combination and may exist on or off the premises.

Public cloud is provided for open use by the general public. This infrastructure may be administered or handled by a business, academic, or government organization or their combination. It exists on the premises of the cloud provider.

Hybrid cloud is composed of two or more distinct cloud infrastructure (private, community, or public), which remain unique entities. They are bound together by standardized or proprietary technology that enables data and application portability.

##### Communication

Regarding eHealth, providing health care services depends on several communication technologies. This is because each choice contains its characteristics, for which providing security requirements is very important. This section can be divided into the 3 subcategories of synchronicity, network design, and connectivity in terms of its details [22].

Synchronicity is employed to coordinate scheduling and technology. Depending on the schedule, telemedicine services can be provided in 2 modes. The first mode is “real-time,” and it refers to a situation in which the people involved in the care and the care providers are related at the same time with each other but in different location situations. The second mode is “store and forward,” and it refers to a donating situation in which the people involved in the care and the care providers are not connected at the same time. Both modes include different technological infrastructure, including video conferencing, telemetry, and remote sensing, as well as other modes of interactive health communication.

Network design/configuration contains the 3 modes of virtual private networks, open internet, and social networks, and in all of these, the information is posted and then shared. To effectively protect the confidentiality of the information of these states, different security settings are required.

Connectivity may be divided into wired and wireless, with different levels of bandwidth and the attendant speed and resolution or quality of service.

#### Dimension 2: Legal

**Figure 5:**
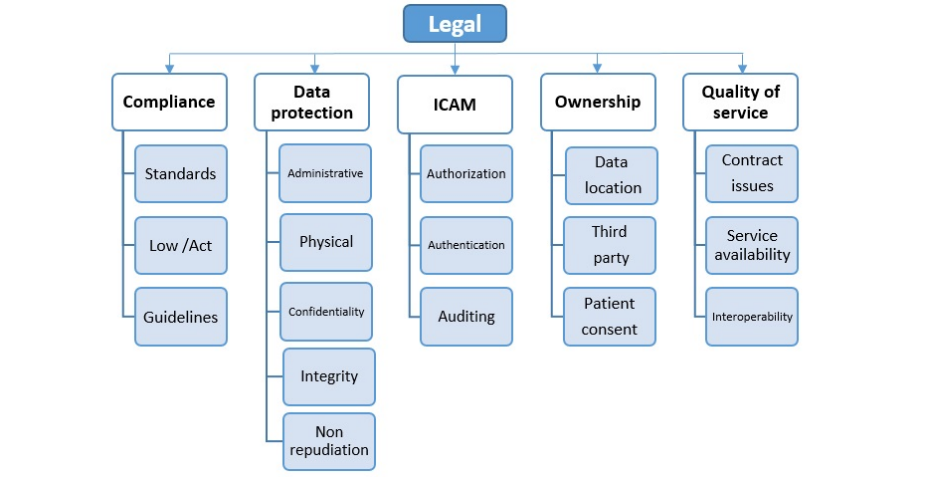
Components of the legal dimension. ICAM: identity credential access management.

According to the assessed studies, the second dimension of this taxonomy is the legal dimension, which can independently provide a framework of legal issues raised in the health cloud. The identified elements of the legal framework have a direct impact on information privacy, which include the 5 main scopes of compliance, data protection, identity credential access management (ICAM), ownership, and quality of service [3]. It should be mentioned that these scopes have a series of subcategories that have been explained in the below text. According to the research findings, privacy and legal issues are completely related and intertwined issues in terms of eHealth. The legal framework scopes are considered as the main components of this dimension (Figure 5).

##### Compliance

The scope of compliance contains the 3 subscopes of standard, law/act/regulation, and policy/guideline [3].

Standard is a document confirmed through consensus by a recognized body that is provided for repeated and common use, and involves rules, guidelines, or characteristics for products or related processes and production methods in which compliance is not mandatory.

Legislation is comparable with statutory law. Legislation restricts the legal requirements as well as the cost or punishment for breaking the law. Most regulations are issued by governments [52].

Policy or guideline is a formal, brief, high-level report or proposal that indicates an organization’s principles, goals, objectives, and acceptable procedures for a topic [3]. Guideline is related to general instructions in order to achieve policy principles. It provides a framework to implement the required procedures.

##### Data Protection

The second scope of this dimension encompasses the details of data protection to provide the technical mechanisms of the requirements introduced in the first scope. Data protection is distributed into the 3 main classes of technical, administrative, and physical issues according to the NIST, Health Insurance Portability and Accountability Act (HIPAA), and Certified Information Systems Security Professional (CISSP) [53].

Technical aims to define supply-related techniques, such as confidentiality, integrity, and nonrepudiation of cloud-based patient data. *Confidentiality* is the guaranteeing process that makes data property or information available or accessible only for authorized people or processes [54]. *Integrity* is the property to ensure the prevention of data or information tampering in an unauthorized manner. *Nonrepudiation* involves service guarantees to make an action taken undeniable.

Administrative involves security infrastructure with a management and development approach, and the implementation and support of systems are discussed [38].

Physical measures policies and procedures to protect the electronic information systems of an entity and the related buildings and equipment from natural and environmental hazards as well as unauthorized intrusion [53].

##### Identity Credential Access Management

The third scope of the second dimension includes data access management, which is a key factor in patients’ rights and medical ethics. Some pertinent fields like identification and authentication, authorization and access control, auditing and monitoring, and user training issues are also placed in this scope. This is a process in which a unique identity is defined for the person or system [53]. It is known as the first step in the access control process, such that it controls any activity based on the identity or entity of the user.

The process of identification and authentication identifies and authenticates the user, which is possible based on the elements and private data created by the user [53].

Authorization is the process of defining the resources and the level of access for the user [53].

System monitoring or auditing is the last loop of this cycle that plays an important role in recording the log of all the activities, events, and performances of the users who have access. Moreover, it is considered a security check [55], which is very important to identify problems and violations with accounts, access, information disclosure, and system operation.

##### Data Ownership

The fourth scope of this dimension is related to data ownership, which is responsible for concepts such as information ownership and responsibility. Information control not only speaks about the creation, modification, and other convolutional procedures of data, but also deals with the rights of individuals to grant or revoke their access to others [12].

The ownership of data in the cloud may rely on the nature of the stored data [12]. Data owners must be able to assess, control, and restrict their data during storage, use, and disclosure [56,57]. Nevertheless, the existing shortcomings in the implementation of these statements in the cloud are considered as some of the essential problems for implementing the cloud in the health sector [57]. This scope encompasses some subscopes like data location issues, third party issues, and patient consent.

Data location involves the storage of data. One of the points in the cloud is that data storage can be carried out in any places, even unknown ones.

Patient consent is derived from the ethical and basic principles of human and citizenship rights in terms of the patient’s discretion [58,59]. In this regard, the patient has the freedom to decide whether the tests and surgeries on the organs can be performed before any action [59-61].

Third party is considered as a cloud provider that does not have any role in the patient’s treatment process as a beneficiary. Nevertheless, it has access to all patient information that can cause several legal dilemmas.

##### Quality of Service

In the fifth scope of this dimension, some issues, such as contract, service availability, and interoperability, are stated, and this has been referred to as quality of service (QoS). It defines guaranteed levels of performance, availability, reliability, interoperability, throughput, performance, response time, etc, all of which are regarded as major factors inﬂuencing the quality of service in cloud computing [62].

Contract issues involve a service level agreement (SLA). This is a mutual agreement between cloud service providers (CSPs) and end users. Quality of service management systems monitor resources, storage, networks, virtual machines, service migration, and fault tolerance [63-65].

Availability involves principles ensuring that authorized users at a proper time have access to the data [53].

Interoperability involves the ability of the system to render services using multiple service providers while preserving the integrity of the data. This feature can be used for all kinds of clouds so that if migration to a different system is required, it can be seamlessly carried out [63,64].

**Figure 6:**
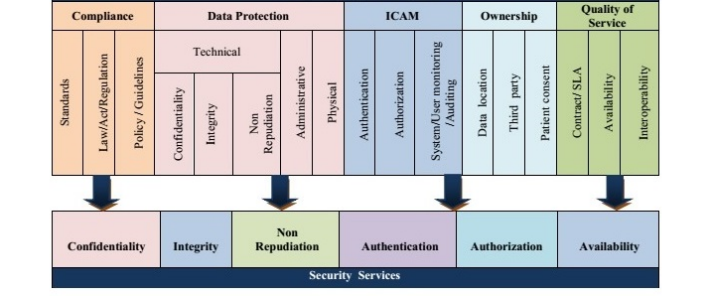
Compatibility between legal frameworks in a security service. ICAM: identity credential access management; SLA: service level agreements.

Figure 6 illustrates the coverage of information security services by legal dimension elements in privacy taxonomy. It is impossible to preserve privacy without considering information security services in dynamic environments, such as the cloud, as these services can ensure benefits in terms of outsourcing the health records [3].

#### Dimension 3: Data

**Figure 7:**
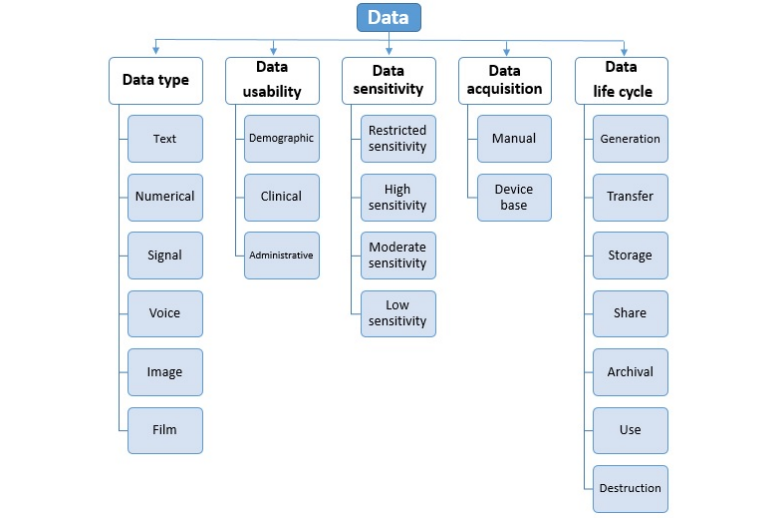
Components of the data dimension.

Data structures are critical in various cloud environments, such as data storage features, data processing methods, and data preserving solutions, designed for this dynamic ecosystem. The third major dimension of our proposed privacy taxonomy is related to data characteristics, which have been divided into the 5 subcategories of data type, data life cycle, data usability, data sensitivity, and data acquisition methods. Figure 7 depicts the structure of the data dimension, although the components of this dimension have an indirect effect on privacy.

##### Data Type

Any data related to health conditions, reproductive outcomes, causes of death, and quality of life are health data [66].

It is worthwhile to mention that health data can measure several criteria, such as clinical, environmental, and socioeconomic factors, both at the individual and population levels, including information about a person’s behavior related to his or her wellness. The accumulation of collected and utilized health data occurs when interacting with health care organizations. The collected data typically contain the received service types, the results of those services, and the clinical outputs or information included in those services.

Health data can be classified into 2 structured or unstructured types. The structured type is a standard that can be simply exchanged between health information systems [66]. For example, a patient’s name, date of birth, or blood test result can be recorded in a structured data format. However, unstructured health data are not standard, unlike the structured type. Emails, audio recordings, or physician notes about a patient are examples of unstructured health data.

Advances in the digital world have improved the collection and use of health data and the databases in the health care industry, which have certain complexities. Overall, in terms of health and care, the data can be classified based on the data type as follows [21]: alphabetical data/textual data/narrative data, numerical data/measurements/coded data, signal data, images/graphic data/pictures, voice, and videos/film.

##### Data Life Cycle

The second scope of the third dimension in the designed privacy taxonomy is data life cycle, which contains 7 phases [67], each including its requirements for privacy. This cycle encompasses the following phases: data generation, data transmission, data storage, data access, data reuse, data archiving, and data disposal. Data life cycle is comparable with the cloud requirements [68-70].

Data generation involves CSPs receiving requests from their users to generate the related data so that they can assign their access control policies.

Data transmission involves CSPs generating a secure transmission channel to verify user data reliability. Besides, they use encryption methods and the digital certificate mechanism between servers.

Data storage involves the role of CSPs to ensure the conformity of the data in the right place according to the agreements and rules.

Data access involves the CSPs ensuring the validity of users’ identity to protect them from spoofing and verifying the proper execution of the data access policy.

Data reuse can lead to leakage of sensitive or personal data, which is a reason for not providing services in the cloud. In the big data era, data sharing has made this phase quite primitive.

Data archiving involves 3 main operations, including band encryption, long-range storage, and data retrieval.

Data disposal is mainly aimed at placing the data completely and effectively in the cloud and removing unnecessary parts.

##### Medical Data Usability

Medical data have very diverse functions, including personal interests, public health, medical research, and development [21]. The use of the data in applications is categorized into 2 modes of primary and secondary. *Primary* is a state where the collected medical data are employed to provide medical care. *Secondary* is a state where the collected medical data are employed for purposes except care.

Here, it is worth noting that digitization and updating based on medical information technology have increased the use of medical data at both primary and secondary levels [21,71]. The data in the patient’s medical file appear in 1 of the following 3 formats based on their origin and applications: *demographic data* (identification data/date of birth, admission, discharge, biometric identifiers, phone number, and health record number); *clinical data* (clinical results/images/summaries, medical data, case management, public health data, performance data, and referral management); and *administrative data* (insurance documents/financial information and nonclinical data focused on record keeping surrounding a service, such as hospital discharge information; it can be part of an electronic health record as well; claims data, which include information regarding insurance claims).

##### Data Sensitivity

One of the important points in privacy preservation is the grading of data regarding their degree of importance. It is performed according to data sensitivity to classify the data based on their sensitivity and the extent of their impact on the patient and the health organization. Accordingly, these importance-based data cannot be disclosed, changed, or destroyed without permission. Classification of the database helps to specify the level of security required by the data. The data are categorized based on their importance level as presented below [72].

Restricted sensitivity of data involves a situation where the data have high sensitivity (restricted sensitivity), and unauthorized access and disclosure of the data may result in significant risks, leading to severe or disastrous adverse effects on the operations and assets of an organization or individual, particularly a patient or health care institution. This level of sensitivity needs the highest level of security controls that must be applied to restricted data.

High sensitivity of data involves a situation where the data have high sensitivity, and unauthorized access and disclosure of the data may alter or destroy the data, leading to serious adverse effects on the operations and assets of an organization or individual, particularly a patient or health care provider. This level of sensitivity needs a reasonable level of security controls that should be applied to private data.

Moderate sensitivity of data involves a situation where the data have moderate sensitivity, such that unauthorized access and disclosure, alteration, or destruction of the data would result in moderate risks for the operations and assets of an organization or individual, especially a patient or health care institution.

Low sensitivity of data involves a situation where the data have less sensitivity, and unauthorized access and disclosure, including alteration or destruction of the data, would lead to a limited risk to the operations and assets of an organization or individual, especially a patient or health care institution, or there will not be any risks.

##### Data Acquisition Methods

When emerging health services arise from the context of modern technologies, such as the cloud, mobiles, wireless multimedia sensor networks (WMSNs), and Internet of Things (IoT), some new scenarios are raised for health care services. These scenarios consist of patient care in hospitals, patient care at home, and self-care scenarios, with each representing a special type. Hence, the protection of data privacy in each scenario requires its characteristics. The important point in terms of privacy preservation in any of these scenarios is to know how to collect the data. Overall, there are 2 collection methods in all these scenarios [21].

In the *manual* method, data are described subjectively or objectively by the patient and then inferred by health care providers. Then, these data are entered into health information systems manually through personal portals*.* In the *device base* method, several medical devices (either wired or wireless) collect data. Subsequently, the collected data are sent to applications for processing to be used by health care providers. Evidently, different types of devices will be fully described in the next section since they play substantial roles in ensuring privacy.

#### Dimension 4: Device

The last dimension identified for the taxonomy of privacy is concerned with devices and their features because, with the advancement of technology, data collection is practically entrusted to devices. Thus, ensuring data privacy is the most important concern of stakeholders in terms of diversity of use.

**Figure 8:**
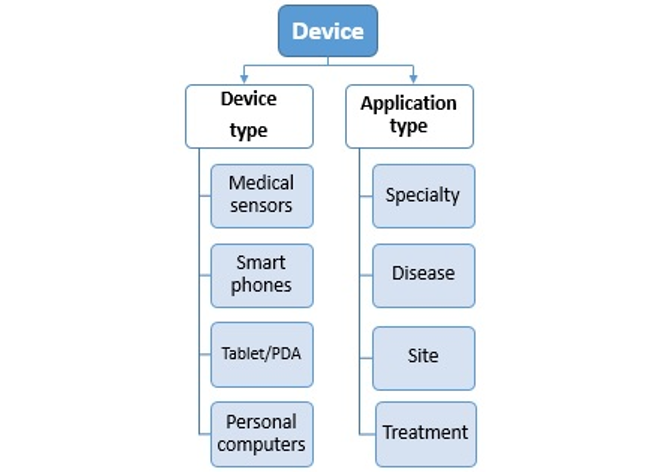
Components of the device dimension. PDA: personal digital assistant.

A medical device is an outfit used to evaluate or diagnose a medical condition [61], for example, electrocardiography machines, ultrasound machines, x-ray machines, different sensors, wireless sensors, and mobile health apps that run on smartphones. Ensuring data privacy on these devices has been an issue in many studies, which makes it challenging in terms of the cloud. As a result, regarding privacy in the cloud, it is essential to consider the features of medical devices. Certainly, the elements defined in this section will have an indirect impact on information privacy in the health cloud. As shown in Figure 8, the device dimension is divided into 2 subcategories: device types and application types.

##### Device Types

WMSNs involve wireless sensors, which are some of the most common devices in the medical world. It is considered as the smallest network and has unique features such as large-scale implementation, portability, and reliability [73]. It should be mentioned that the sensor network encompasses a set of independent nodes with low cost, energy, and memory, and limited computing power [73]. The health care industry has experienced a dramatic transformation with the use of WMSNs [74]. The main aim of WMSNs is to collect and transfer environmental data to central databases or remote locations. IoT is another popular tool in recent years [65,75], which has created a new technological paradigm in the health care industry. In eHealth, IoT has provided the possibility of interaction and communication between “things” via the internet. In future health care circumstances, IoT will connect subjects and health care professionals seamlessly [76,77].

These technologies can be used for eHealth applications, such as computer-assisted rehabilitation, early detection of medical issues, and emergency notifications. However, there is an issue because several factors limit the use of these technologies. The most important factor is legal issues related to the privacy and security of the data transmitted [78-81].

Smartphones have become an integral part of life. Thus, they can act as a gateway between the wireless body area network (WBAN) and IoT [82-84]. Essentially, the smartphone’s sensor data or high-resolution camera images are sampled, processed into medical information, and displayed [84]. Using smartphones for medical purposes can be very useful because millions of people have their own smartphones today and can access medical applications designed for health care [61].

Tablets/personal digital assistants have the same applications as smartphones, acting as a gateway to collect medical data beyond providing accessibility to reference textbooks [85].

Personal computers play a pivotal role in information management. Computers potentially alter the traditional approach that physicians use to communicate with patients [86] and have an essential role in information management. In other words, they can change the traditional ways of providing health services to patients and replace them with novel innovative methods [86].

All of the above-mentioned tools with increasing use in medicine must comply with certain features to ensure the privacy of data since ignoring these features can cause some irreparable damage.

##### Application Types

Care processes across virtually all basic medical specialties and subspecializations associated with disease entities, sites of care, and treatment modalities are included. The vast array of these applications and the complexity of the medical practice and medical specialization are listed separately [22]. The second device subcategory is related to application types.

Basic specialties include content areas around specific diseases, including diabetes, stroke, and posttraumatic stress disorder, and such applications have been developed. Moreover, programs may differ by the site of care, including the intensive care unit, outpatient psychiatry unit, emergency department, and home. Some programs were organized around specific treatment modalities such as rehabilitation and pharmacy. Over 40,000 health applications have been used on smartphones [61]. The World Health Organization has classified mobile health applications as follows [70]: toll-free emergency, health call centers, public health emergencies, mobile telemedicine, information initiatives, appointment reminders, community mobilization, treatment compliance, patient records, surveillance, health surveys, patient monitoring, decision support systems, and awareness raising [20]. Depending on the site of care, these applications have several privacy requirements that must be identified and met. In other words, the privacy of a user’s data in the devices depends on the security of the designed computer programs.

### Comparisons to Existing Literature

From these dimensions, it is understandable that the *legal* dimension and its subcomponents have direct influence on privacy and other dimensions like *data*, *device*, and *cloud* along with their subcomponents, as well as an impact on privacy preservation concerns in the cloud environment.

In contrast with other taxonomies, this taxonomy sides with health data specification and cloud considerations, which appear critical. Therefore, this article first tries to adopt the privacy taxonomy in the cloud context, especially in the health cloud, and the remainder is dedicated to redefining privacy terms with new details. 

The health care domain has the most complicated scenarios and most varied data among businesses. Thus, when a taxonomy fits with its requirements, the taxonomy might be appropriate for other domains, businesses, and scenarios that are complex. In fact, the user of the model should exercise judgment as to the appropriate level of detail necessary to test the target hypothesis. 

### Usability and Experimental Use of This Taxonomy

This well-organized taxonomy has some advantages like presenting the hierarchical root of concepts and inherited features of taxonomies. It provides a heuristic representation of hierarchies with 4 dimensions of privacy and the branches of each dimension. This model allows for more specification of independent variables in model development and with regard to research objectives. Experimental use of this taxonomy depends on the following stages: scenario clarification stage, device and system specification stage, data specification stage, and privacy mapping stage.

In the first stage, the specification of cloud-based scenarios should be clarified. For example, which service model and cloud deployment have been chosen for health care delivery and which communication method has been chosen to connect the stakeholders individually or with each other (synchronized or unsynchronized; wired or wireless).

In the second stage, the use of medical devices and application types for data collection should be prominent and transparent to users because each device has its specific privacy requirements.

In the third stage, data specifications collected in each scenario should be explicated because the veracity in data specifications can lead to variations in privacy strategies. For instance, in one scenario, electrocardiography data detected by the WMSN and transferred via a designated mobile health app to the cloud for storage, processing, and use will have special privacy requirements. In another self-care scenario, subjective data that are just entered through a cloud-based personalized portal need a different set of privacy requirements.

In the fourth stage, to ensure privacy preservation in all means, the identified features in other stages should match with legal components from the proposed taxonomy. For example, proper corresponding security services like authentication, authorization, auditing, confidentiality methods, integrity, and nonrepudiation methods should be chosen for each type of health care scenario in the digital world. Through these approaches, stakeholders can trust eHealth.

This taxonomy generally has 2 layers of stakeholders (people and organizations, and applications and systems).

The first layer involves people and organizations, including patients; health cloud and general cloud providers; health care providers (eg, physicians and nurses); health care organizations (eg, hospitals, laboratories, drug stores, and physicians’ offices); cloud app developers and vendors; health domain stakeholders (eg, insurance companies and financial organizations); researchers and practitioners working in areas like health, cloud, data management, security, and privacy; medical ethics authorities; organizations planning to design and deploy cloud services and migrate to cloud platforms and services; governments and legislation bodies; and national or international standardization bodies. These groups, according to the scenario clarification stage, device and system specification stage, and data specification stage, map their privacy preferences with respect to the proposed privacy taxonomy.

The second layer involves applications and systems that are affected by this taxonomy, including patient assessment systems; telemedicine systems; medical imaging systems; public health systems; hospital information systems; clinical information systems; health data secondary use systems; teleconsultation systems; self-care systems; and medical device and wireless system producers (WMSN, IoT, etc). These systems by their provisions can meet privacy requirements according to the proposed privacy taxonomy.

Considering the above-mentioned stakeholders, among the main approaches to deal with privacy challenges, identifying the contributing factors and dimensions can be helpful to manage this domain.

### Limitations of the Study and Future Work

This study has some limitations. The interchangeable use of some related terms like “security,” “privacy,” and “legal” made the close assessment of articles difficult, and it was challenging to obtain findings from related comprehensive articles with regard to health industry scenarios.

An attempt was made to include English papers; therefore, the results must be considered within the scope of the English literature and studies in a specific interval. Any papers published before or after the search interval were not included; however, there is always the possibility of missing some relevant information or bias.

Future studies can be conducted to identify or propose definite standards and requirements for privacy preservation in each subcategory of known dimensions. It is hoped that the proposed taxonomy will not only clarify nomenclature proliferation in privacy for the health cloud or eHealth, but also provide a useful guide for research and policy making.

This taxonomy is not a finished product and needs more attention with regard to development and improvement. The process has been initiated with the hope that others in the field will be interested in it and complement the privacy taxonomy in the health cloud. Furthermore, this taxonomy can be considered as the subject matter for experts in various domains of privacy for assessment, testing, revision, and verification.

### Conclusion

This research was conducted to identify the factors affecting privacy in the health cloud and classify them to provide a unique and comprehensive taxonomy through the investigation of related papers. It redefines the health cloud privacy term by using a deductive approach.

The proposed taxonomy tries to provide the true and full perspectives of the intervention, management, and handling of other variables, as well as itemize the expected outcomes and determine how best to assess them, thus clarifying the units of analysis in health cloud privacy research.

The subscribed elements have been classified into the 4 main dimensions of *cloud*, *legal*, *data*, and *device*. Moreover, since taxonomy designing is an iterative process, 15 components and 57 elements were added to these 4 main dimensions in 3 layers.

Among all these elements, those classified in the legal dimension had a direct impact on data privacy in the cloud. However, other elements will have an indirect impact on ensuring data privacy in the cloud.

In the second step, this taxonomy tried to clarify the privacy concept in eHealth, which is a multidisciplinary context, and tried to remove the ambiguities between existing definitions in the field of security and define a clear boundary for the words. This led to the distinction and clarification of the overlapping and vague structure of related concepts, and privacy was defined by identifying the discrete sets of variables representing specific privacy configurations and definitive boundaries for “security,” “privacy,” and “legal” terms, which are crucial for future research, policy making, and the actual management of privacy. Therefore, users can have a more accurate definition of the concepts in this field in the future.

This taxonomy is designed to satisfy the needs of emerging technologies, such as mobile health, health IoT, telemedicine, etc, which use cloud devices in their infrastructure. Moreover, it can be considered as supplementary classification and a reference for current privacy, security, or technological taxonomies.

Hence, this taxonomy can cover health industry requirements with its specifications like health data and scenarios, which are considered as the most complicated among businesses and industries. Therefore, the use of this taxonomy could be generalized and customized to other domains and businesses that have less complications.

This paper has also reviewed the most popular previous taxonomies in the privacy domain.

## Abbreviations

CSP: cloud service provider
IoT: Internet of Things
NIST: National Institute of Standards and Technology
PRISMA: Preferred Reporting Items for Systematic Reviews and Meta-Analyses
WMSN: wireless multimedia sensor network
